# *In situ* remineralisation response of different artificial caries-like enamel lesions to home-care and professional fluoride treatments

**DOI:** 10.1186/s12903-016-0160-9

**Published:** 2016-01-08

**Authors:** Priscila Maria Aranda Salomão, Lívia Picchi Comar, Marília Afonso Rabelo Buzalaf, Ana Carolina Magalhães

**Affiliations:** Department of Biological Sciences, Bauru School of Dentistry - University of São Paulo, Al. Dr. Octávio Pinheiro Brisolla 9-75, Bauru, SP 17012-901 Brazil

**Keywords:** Demineralisation, Enamel, Fluoride, In situ study, Remineralisation

## Abstract

**Background:**

Artificial lesions produced by different protocols might directly influence the response to different remineralising treatments. This study compared the response of different artificial caries-like enamel lesions to home-care and professional fluoride based-remineralising treatments *in situ*.

**Methods:**

The tested demineralising protocols were methylcellulose- MC gel, polyacrylic acid - PA gel, tetraethyl methylene diphosphanate - TEMDP solution, and acetate- Buffer solution. The lesions were remineralised using an *in situ* model, following a crossover and double blind design. Twelve subjects wore intra-oral appliances during 3 phases (3 d each): control (C) (saliva); home-care F^−^ treatment (FD) (1,100 ppm F^−^ dentifrice, 2x1 min/day); and professional (FVD) (22,600 ppm F^−^ varnish) plus FD. The de-remineralisation was measured by transverse microradiography-TMR and hardness (surface hardness/cross-sectional hardness, SH/CSH, respectively).

**Results:**

For SH, lesions produced by PA gel were the only one showing significant differences among the remineralising treatments (C x FD x FVD); while the TEMDP lesion were not responsive to any fluoride treatment (for both SH/CSH). For TMR, there were no differences among the remineralising treatments, regardless of the type of lesion. Generally, the most responsive lesions to fluoride were the less demineralised lesions (considering hardness: PA gel and Buffer).

**Conclusions:**

The type of lesion has influence on the surface remineralisation degree induced by home-care and professional fluoride treatments using this *in situ* model.

## Background

Enamel caries lesion begins initially as a surface-softened demineralisation, progressing to a subsurface lesion over time, which is clinically seen as a white-spot lesion [1]. *In vitro* protocols should be able to produce artificial lesions as close as possible to the *in vivo* condition. However, the artificial lesions produced by different laboratory protocols significantly differ in respect to mineral distribution and depth [2–6]. Those differences should be considered in the study design, as they might directly influence the response to further remineralising treatments [2, 5, 7–9].

In a previous study, four protocols to produce artificial caries-like enamel lesions (methylcellulose- MC gel; polyacrylic acid - PA gel; tetraethyl methylene diphosphanate - TEMDP solution and acetate - Buffer solution) were compared, from which TEMDP induced the highest subsurface mineral loss and depth [4]. However, there is no comparative information about the behaviour of those lesions when subjected to remineralisation.

Some works have investigated the response of different lesions to remineralising agents such as fluoride, however, they have tested one or two types of artificial caries-like lesions, varying the time of demineralisation rather than comparing different published demineralising protocols [3, 5, 8, 9].

Lippert et al. [5] compared the response of two types of lesions with different baseline mineral distributions (R) to fluoride (1,100 ppm F^−^) treatment *in situ*. Low-R (mineral loss average) lesions further demineralised, whereas high-R lesions exhibited some remineralisation. This finding highlights the importance of studying the response of different type of lesions to remineralisation. However, the former authors tested only the remineralising effect of fluoride dentifrice. It would be interesting to know the response of the artificial caries-like lesions to different remineralising treatments, one simulating the home-care fluoride application (as dentifrice) and other in combination with professional fluoride application (as varnish).

Transversal microradiography (TMR) and cross-sectional micro-hardness (CSH) are useful methods to analyse de-remineralisation. Recent comparative data from TMR and CSH measurements showed that the relationship between mineral content and hardness is not linear for demineralised enamel, and this relationship is highly dependent on the type of lesion [4, 10]. However, there is no information about the relationship between both variables on demineralised enamel subjected to different remineralising agents.

Therefore, the study aimed firstly to compare the response of the artificial caries-like enamel lesions produced by four different *in vitro* protocols to the remineralisation induced by control-saliva only (C); home-care F^−^ treatment (dentifrice, FD); or professional (varnish) plus home-care F^−^ treatments (FVD) *in situ*. The study also aimed to check if there is any correlation between CSH and TMR data for both demineralised and de-remineralised enamel. For these purposes, the null hypotheses tested were: 1) There is no difference among the artificial lesions regardless of the remineralising protocols. 2) There is no difference among the remineralising protocols regardless of the type of artificial lesion. 3) there is no correlation between CSH and TMR for demineralised enamel. 4) there is no correlation between the CSH and TMR for de-remineralised enamel.

## Methods

### Ethical aspects and experimental in situ design

Ethical approval for the study, involving human subjects, was granted by the “Ethical Committee in research involving Humans” from Bauru School of Dentistry-University of São Paulo (Process n° 036-2011). Written and signed consent was obtained from all subjects before starting the study.

Twelve healthy adults (18–30 years of age) were enrolled according to the study inclusion and exclusion criteria [11]. Inclusion criteria were defined as follows: a stimulated physiological salivary flow rate of >1 ml.min^−1^; a non-stimulated physiological salivary flow rate of >0.25 ml.min^−1^; and good oral health (i.e. no cavities or significant gingivitis/periodontitis). Exclusion criteria were: systemic illness; pregnancy or breastfeeding; use of fixed or removable orthodontic appliances; use of fluoride mouthrinse or professional fluoride application in the last 2 months. A sample size of 12 subjects was considered according to a previous *in situ* study comparing fluoride and placebo varnishes on enamel caries remineralisation (% surface hardness recovery, %SHR), considering α-error level of 5 % and β-error level of 20 % [11].

This prospective, randomised, crossover, and double-blind (the author PMAS and the subjects) study was performed with a washout interval of 7 d before each of the 3 experimental phases of 3 d duration [12]. The experimental treatments/phases were as follows: control (C) - saliva effect only (placebo dentifrice); home-care (FD) (fluoride dentifrice: 1,100 ppm F^−^, NaF); and professional (varnish: 22,600 ppm F^−^, NaF, prior the *in situ* study) plus home-care fluoride applications (FVD). The subjects were randomly allocated to each experimental phase.

At the first phase, four subjects took part on treatment C, four on treatment FD and the other four on treatment FVD. At the other phases, the enamel samples and the treatments were changed, in order to provide a real crossover design, in which all subjects were randomly subjected to all treatments.

### Specimen preparation and demineralisation procedures

Enamel specimens (4 mm X 4 mm X 2.5 mm, n = 288) were prepared from bovine incisors as previously described [11]. The polished specimens were allocated to 4 demineralising protocols by stratified randomisation according to their baseline surface hardness (SH) means (n = 72): 1) MC gel; 2) PA gel; 3) TEMDP solution; and 4) Buffer solution. The solutions and gels were prepared according to Magalhães et al. [4] (Table 1).

**Table 1:** Description of the demineralising protocols applied *in vitro*

Demineralisation*		pH	Period
MC Gel	8 % methylcellulose gel layer of 0.5 cm at 4 °C. After 12 h, covered with an equal volume (1.5 mL) of 0.1 M lactic acid.	pH 4.6	14 days
PA Gel	20 g/L Carbopol 907 (PA, molecular weight 450,000 Da), 500 mg/L hydroxyapatite and 0.1 M lactic acid.	pH 4.8	16 h
	25 mL gel/ specimen		
TEMDP Solution	3 mM CaCl_2_ · 2H_2_O (LabSynth), 3 mM KH_2_PO_4_ (Sigma-Aldrich), 50 mM lactic acid (Sigma-Aldrich), 6 μM TEMDP (Sigma-Aldrich); traces of thymol.	pH 5.0	6 days
	30 mL solution/ specimen.		
Buffer Solution	1.28 mM Ca(NO_3_)_2_ · 4H_2_O (Merck), 0.74 mM NaH_2_PO_4_ · 2H_2_O (LabSynth), 50 mM glacial acetic acid and 0.03 ppm F (NaF).	pH 5.0	16 h
	30 mL solution/specimen		

Footnote: * All demineralising protocols were done at 37 °C

Nail varnish was applied on 1 outer third of the enamel surface (unexposed area, sound enamel surface), and then the specimens were individually immersed in unstirred acidic solutions or gels at 37 °C. After demineralisation, the specimens were washed and dried. The other outer third enamel surface was protected with nail varnish (demineralised enamel surface). According to the enamel hardness loss values (%SHC – surface hardness change), the specimens were first randomly assigned to the *in situ* phases (remineralising treatments), then to the subjects and to the places in the appliance, by using Excel Microsoft.

### In situ remineralising treatments

Four cavities 10 mm wide X 10 mm wide X 3 mm deep were made, two on each side (left and right sides) of the acrylic palatal appliance. In each cavity, two enamel specimens corresponding to one of the demineralising protocols were fixed with wax. For each phase, a new appliance was made, and the specimens were replaced. The specimens were placed at the same level of the acrylic, so no biofilm accumulation was allowed.

During the 3 d phase, the appliance was only removed during the main meals (4 times a day, maximum of 1 h duration each, interval between meals of 2–3 h). Immediately after the meals, before reinserting the appliance in the mouth, the subjects were advised to perform oral hygiene using dentifrice according to the treatment phase (treatment C: non-fluoridated from IceFresh® or treatments FD and FVD: fluoridated dentifrice from Crest-Procter & Gamble®, Cincinnati, USA).

The application of dentifrice (all phases) was completed for 1 min twice a day (after the first and last oral hygiene of the day) [5], by applying slurry of the dentifrice (1:3 water, 1 drop/specimen) on the specimens. Thereafter, the appliance was reinserted in the mouth, and the subjects were advised to rinse with 10 ml of water for 5 s and, then, to expectorate.

In respect to the professional treatment, the varnish (Duraphat, Colgate®, São Bernardo do Campo, Brazil) was applied prior the *in situ* study, using a microbrush. Thereafter, the specimens were stored in remineralising solution [13], at 25 °C. After 6 h, the varnishes were removed [14], and the specimens were placed in the appliance.

The subjects were extensively trained for the correct execution of the study and were also advised not to eat or to drink while the appliances were in the mouth and not to use any fluoride or/and antiplaque agents.

### Hardness measurement

Surface knoop hardness was measured at baseline (SH baseline), after demineralisation (SH lesion) and at the end of the *in situ* remineralisation (SH final), 100 μm apart from each other, on the central area (5 indentations, 25 g, 10s), using a microhardness tester (Buehler Micromet 5114, Lake Bluff, USA). The percentage of surface hardness change (SHC) was calculated after demineralisation [% SHC = 100 - (SH lesion*100/SH baseline)] and the percentage of surface hardness recovery (SHR) was calculated after remineralisation [% SHR = (SH final – SH lesion)/(SH baseline–SH lesion) X 100].

Thereafter, the specimens were perpendicularly sectioned to the central band, and one half per specimen was analysed using cross-sectional hardness (CSH); the other half was prepared for the transverse microradiography (TMR).

The specimen was embedded in acrylic resin and polished as described previously [11]. Three rows of 8 indentations each were made on the de-remineralised enamel area (CSH final), and two rows of 8 indentations each were made on sound and demineralised enamel areas (CSH baseline and CSH lesion, respectively). The indentations were made at 10, 30, 50, 70, 90, 110, 220, and 330 μm from the outer enamel surface. The percentage of CSH recovery (%CSHR) at each depth was also calculated as done for the surface hardness.

### Transverse microradiography

Half of the specimen separated for TMR was further sectioned and hand polished to obtain a specimen of an approximate thickness of 100 μm. The fragments were fixed in a sample-holder together with an aluminium calibration step wedge with 11 steps. A microradiograph was taken using an x-ray generator (Softex, Tokyo, Japan) on the glass plate at 20 kV and 20 mA (at a distance of 42 cm) for 20 min. The glass plates were developed for 5 min, rinsed in deionized water, fixed for 3 min in a dark environment, and then rinsed in running water for 10 min and air-dried (all procedures were done at 20 °C). The developed plate was analysed using a transmitted light microscope fitted with a 20x objective (Zeiss, Germany), a CCD camera (Canon, Japan), and a computer. Two images per specimen were taken using data-acquisition (version 2012) and interpreted using calculation (version 2006) softwares from Inspektor Research System bv (Amsterdam, The Netherlands).

The mineral content was calculated based on the work of Angmar et al. [15], assuming the density of the mineral to be 3.15 kg l^−1^ and 87 vol% of mineral content for the sound enamel. The lesion depth (LD), the integrated mineral loss (ΔZ), the average mineral loss over the lesion depth (R), and the mean thickness of the “pseudo-intact” surface layer (SL) were calculated for the demineralised (lesion) and de-remineralised (final) enamel. For the comparison among the remineralising treatments, the differences were calculated as follows: ΔΔZ = ΔZ lesion - ΔZ final and ΔLD = LD lesion – LD final. The % of ΔZ recovery (%ΔZR) was also calculated considering ΔZ lesion as 100 %.

### Statistical analysis

Data was statistically analysed using the GraphPad Instat and GraphPad Prism for Windows (GraphPad Software, San Diego, USA).

ANOVA followed by Tukey’s test were applied to compare the demineralising protocols (CSH lesion, ΔZ, and LD). For the parameters %SHC, R, and SL, no parametric Kruskal-Wallis and the post-hoc Dunn tests were applied, since the data did not reach homogeneity.

To compare the remineralising treatments (%SHR, %CSHR, ΔΔZ, ΔLD and %ΔZR values), two-way repeated measures ANOVA followed by the Bonferroni test were applied, considering as factors: type of lesion and remineralising treatment.

To achieve the second aim of the study, the data from CSH and TMR (% mineral content) at 10, 30, 50, 70, 90, 110, 220, and 330 μm from the outer enamel surface were submitted to linear regression (Statistica, Statsoft, Tulsa, USA). The number of subjects was adopted as the sample size and the significance level for all tests was set at 5 %.

## Results

### Hardness data

The degree of surface demineralisation (%SHC) was higher for TEMDP compared to the other protocols, which did not differ from each other except between MC gel and Buffer. Table 2 shows both the surface demineralisation (%SHC) and remineralisation (%SHR) data. The %SHR at the surface was depending on the treatment. In the presence of fluoride, TEMDP presented the lowest surface remineralisation, while and PA gel and Buffer lesions, the highest one.

**Table 2:** %SHC (lesion) and %SHR/%CSHR (final) values of enamel lesions under different remineralising treatments *in situ*

		%SHR (surface)			%CSHR (10 μm depth)		
	% SHC*	C	FD	FVD	C	FD	FVD
MC Gel	83.8 ± 6.0^A^	10.0 ± 3.1^Ba^	16.1 ± 4.8^Bb^	17.3 ± 6.4^Bb^	4.6 ± 4.7^Aa^	10.1 ± 4.5^ABa^	9.4 ± 6.3^Aa^
PA Gel	84.9 ± 4.1^AB^	9.6 ± 2.2^ABa^	15.2 ± 3.0^Bb^	23.8 ± 4.5^Cc^	4.6 ± 7.9^Aa^	18.2 ± 14.7^Bb^	19.0 ± 6.7^Bb^
TEMDP	95.2 ± 7.1^C^	6.0 ± 2.9^Aa^	8.9 ± 3.7^Aa^	6.8 ± 4.5^Aa^	4.0 ± 3.8^Aa^	4.2 ± 3.6^Aa^	4.8 ± 3.4^Aa^
Buffer	87.1 ± 8.8^B^	12.5 ± 4.4^Ba^	21.9 ± 6.3^Cb^	22.2 ± 7.1^Cb^	3.4 ± 5.6^Aa^	14.8 ± 11.5^Bb^	23.2 ± 22.2^Bb^

Control: C; F^−^ dentifrice: FD; F^−^ varnish and dentifrice: FVD

Footnote: Mean and standard deviation and * median and interquartile range at surface and subsurface

%SHC: Values in the same column with different superscript uppercase letters differ significantly from each other (Kruskal-Wallis followed by Dunn test, p < 0.0001)

%SHR/ %CSHR: Values in the same column with different superscript uppercase letters show significant differences among the lesions. Values in the same line with different superscript lowercase letters show significant differences among the remineralising treatments (two-way repeated measures ANOVA followed by Bonferroni test, p < 0.0001 for lesion and p < 0.001 for treatment)

When the remineralising treatments were compared, %SHR significantly increased after both fluoride treatments compared to the control for all type of lesions, except TEMDP. For MC gel and Buffer lesions, no significant difference was found between FD and FVD. PA gel lesions were the only one demonstrating significant differences between FD and FVD.

**Fig. 1:**
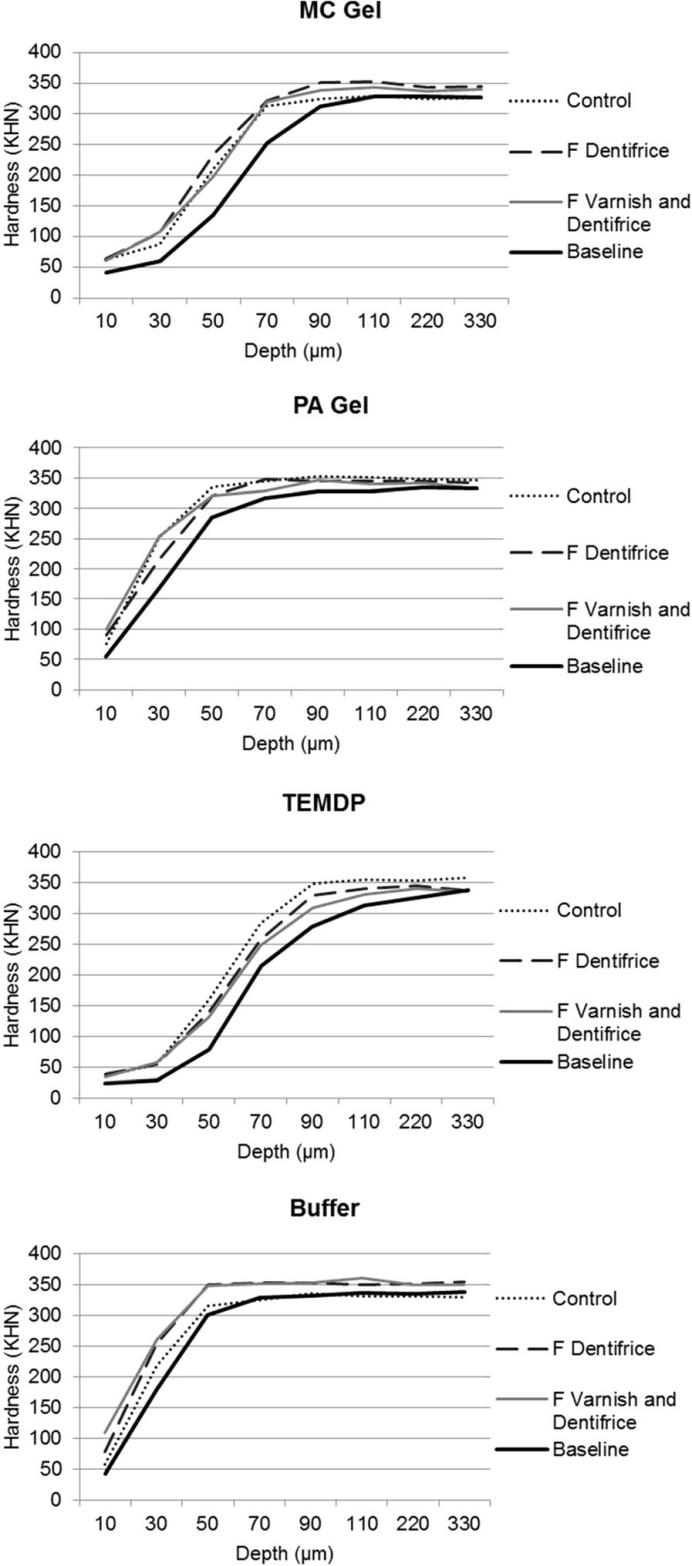
Hardness profile (lesion and final) for each type of lesion and remineralising protocol. Footnote: *Two-way repeated measures ANOVA followed by Bonferroni test (p < 0.0001 for lesion, p < 0.0001 for treatment, p < 0.05 for interaction between factors at 10, 90, 110, and 330 μm and p > 0.05 at 30, 50, 70, and 220 μm)

Figure 1 shows the cross-sectional hardness recovery along to the lesion depth for all type of lesions and treatments compared to the baseline. Table 2 shows the CSH data only at 10 μm depth, since no significant differences were found among the lesions and treatments for depths > 10 μm. In the presence of fluoride, TEMDP presented the lowest remineralisation at 10 μm depth compared to PA gel and Buffer lesions, but did not differ from MC gel lesions. PA gel and Buffer lesions were able to show significant differences between both fluoride treatments (FD and FVD) versus control. No significant differences were found among the remineralising treatments for MC gel and TEMDP lesions (Table 2).

### TMR data

Buffer/PA gel and TEMDP presented the lowest and the highest ΔZ lesion, respectively (Table 3). The surface layer was seen in 100 % of specimens except for those demineralised by using PA gel (only 42 % presented surface layer).

**Table 3:** Baseline TMR parameters for the different artificial carious lesions before remineralisation

	ΔZ	LD	R*	SL*
MC Gel	2452.8 ± 403.7^B^	81.5 ± 12.3^A^	30.0 ± 3.3^AB^	7.1 ± 5.3^A^
PA Gel	1445.9 ± 387.4^C^	54.6 ± 14.2^B^	26.1 ± 8.2^B^	0.2 ± 1.6^B^
TEMDP	2949.8 ± 462.9^A^	81.4 ± 10.7^A^	38.1 ± 6.8^A^	7.1 ± 2.8^A^
Buffer	1349.0 ± 208.9^C^	50.3 ± 11.1^B^	27.8 ± 6.7^B^	1.9 ± 2.0^B^

Footnote: Mean and standard deviation or *median and interquartile range

Values in the same column with different superscript uppercase letters differ significantly from each other. For ΔZ and LD, significance was determined using ANOVA followed by Tukey test (p < 0.0001). For R and SL, significance was determined using Kruskal-Wallis and Dunn tests (p < 0.0002 and p < 0.0001, respectively)

**Fig. 2:**
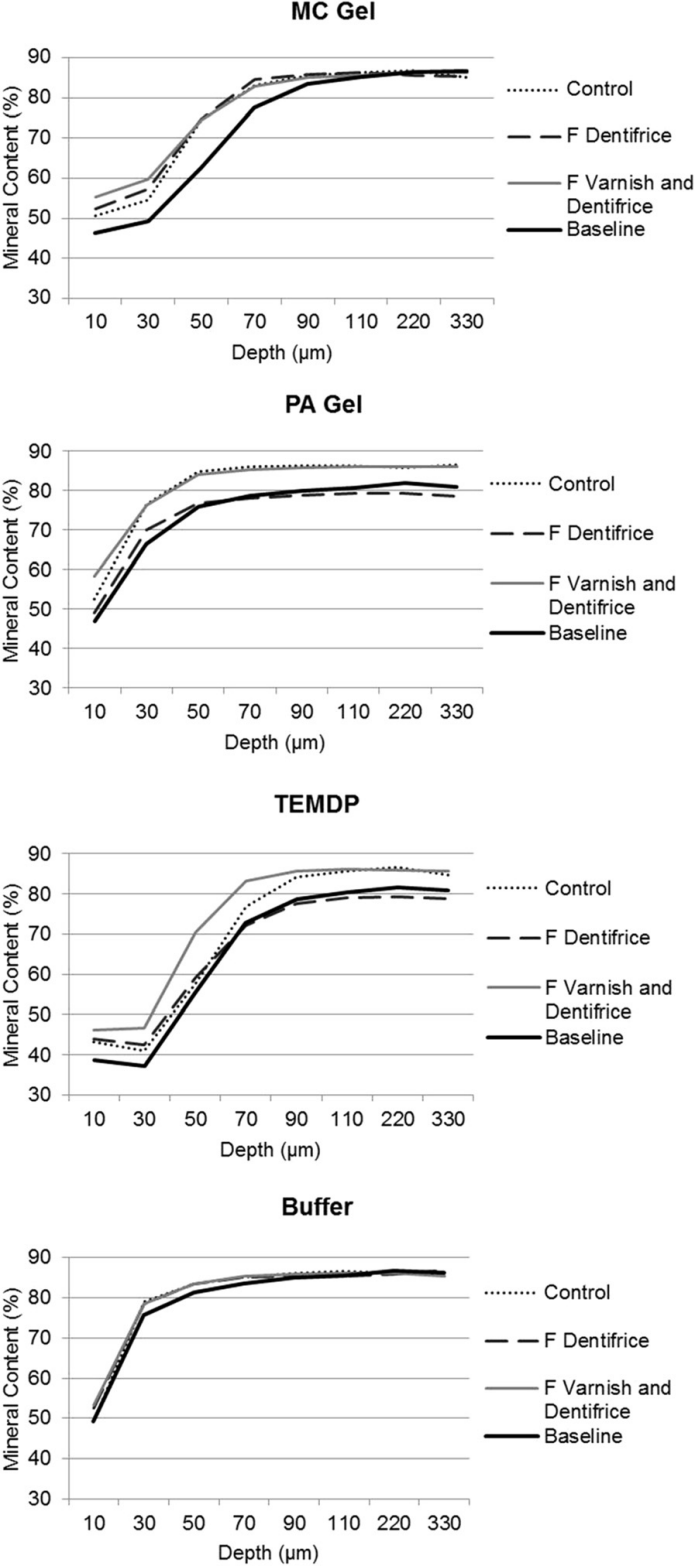
Mineral profile (lesion and final) for each type of lesion and remineralising protocol. Footnote: *Two-way repeated measures ANOVA followed by Bonferroni test (p < 0.01 for lesion, p > 0.05 for treatment, and p > 0.05 for interaction between factors)

Figure 2 shows some mineral recovery along to the lesion depth for all type of lesions, except Buffer that showed a slight remineralisation compared to the baseline mineral profile.

Considering the % ΔZR and ΔLD data, all treatments were similarly able to remineralise enamel lesions, except TEMDP compared to MC gel lesions in control phase. When the remineralising treatments were compared, no significant differences were found among them (Table 4).

**Table 4:** ΔΔZ (%ΔZR) and ΔLD values for enamel lesions under different remineralising treatments *in situ*

	Control		F dentifrice		F Varnish and dentifrice	
	ΔΔZ	ΔLD	ΔΔZ	ΔLD	ΔΔZ	ΔLD
	%minxμm	μm	%minxμm	μm	%minxμm	μm
MC Gel	830.4 ± 626.1^Aa^	19.3 ± 15.0^Aa^	694.2 ± 458.8^Aa^	18.1 ± 17.2^Aa^	713.3 ± 580.7^Aa^	11.5 ± 10.5^Aa^
	(29.6 ± 20.5^Aa^)		(27.8 ± 16.2^Aa^)		(27.7 ± 16.8^Aa^)	
PA Gel	345.6 ± 307.0^Ba^	11.3 ± 19.0^ABa^	336.8 ± 237.3^ABa^	12.5 ± 8.6^Aa^	531.3 ± 629.0^Aa^	15.9 ± 12.4^Aa^
	(22.8 ± 19.9^ABa^)		(22.2 ± 11.7^Aa^)		(29.9 ± 19.3^Aa^)	
TEMDP	295.8 ± 683.8^Ba^	5.0 ± 18.1^Ba^	622.3 ± 451.3^ABa^	15.1 ± 12.9^Aa^	572.1 ± 335.7^Aa^	13.0 ± 12.7^Aa^
	(8.4 ± 19.5^Ba^)		(19.9 ± 11.6^Aa^)		(20.1 ± 10.6^Aa^)	
Buffer	181.7 ± 171.0^Ba^	5.9 ± 5.7^ABa^	223.8 ± 322.1^Ba^	10.9 ± 10.5^Aa^	447.9 ± 367.2^Aa^	2.9 ± 15.4^Aa^
	(14.3 ± 11.6^ABa^)		(14.5 ± 32.5^Aa^)		(27.1 ± 12.4^Aa^)	

Footnote: Mean and standard deviation. Values in the same column with different superscript uppercase letters show significant differences among the lesions. Values in the same line with different superscript lowercase letters show significant differences among the remineralising treatments for each TMR parameter separately

Two-way repeated measures ANOVA followed by Bonferroni test, p < 0.05 for lesion, p > 0.05 for treatment, and p > 0.05 for interaction between factors

### Relationships between hardness and mineral content

A strong coefficient of determination was observed between both variables for demineralised and de-remineralised enamel (r^2^ > 0.9 and r^2^ > 0.95, respectively, p < 0.01). The regression formulas found for demineralised (1) and de-remineralised (2) enamel were, respectively: (1) %min = 40.70 + 0.12xCSH + 3.82xCSH(MC gel) + 4.46xCSH(buffer) and (2) %min = 42.93 + 0.12xCSH + 3.25xCSH(MC gel) + 1.95xCSH(buffer) – 3.55xCSH(F dentifrice).

## Discussion

Artificial caries-like enamel lesions, with differences in mineral distribution and depth, may have a distinct behaviour when submitted to remineralising treatments [2, 5, 8, 9]. Therefore, this paper presents two factors under study: four different artificial caries-like enamel lesions and three remineralising treatments that were applied on these lesions *in situ,* to simulate the oral environment [16]. The remineralising treatments included: saliva only (as control); fluoride dentifrice (home-care); and fluoride varnish (professional) plus fluoride dentifrice (home-care) treatments.

One of the lesions was produced by using MC gel [17]. This protocol is simple to be done, due to the low number of reagents needed to prepare this demineralising medium. However, the lesion is not homogeneous, which might be due to the consistency of methylcellulose gel precluding uniform penetration of lactic acid on the enamel surface. Lippert et al. [18] have shown that the gel viscosity may have an impact on the production of carious lesions - more viscous gels tend to produce less demineralised lesions. The MC gel lesions produced in our study showed a good reproducibility (considering TMR data) compared to those done by ten Cate et al. [17]. However, only the mineral distribution (R values) of our MC gel lesions was similar to previous study by Lynch and ten Cate [2], which could be due to some differences in the gel viscosity or purity.

Different from MC gel, PA gel lesions [19, 20] produced in this study were similar to those found in a previous study [4]. However, the PA lesions were less demineralised (considering TMR data) than those produced by White [19] and Iijima et al. [20]. Taking into consideration all the comparisons, MC and PA gel protocols seem to be partly reproducible among the different laboratories. It is also important to point out that only 42 % of lesions created by PA gel were typical subsurface lesions.

With respect to the TEMDP lesions [21], the protocol induced highly demineralised enamel, making it difficult to measure the hardness at the surface. Using acetic acid, Buskes et al. [21] induced a deeper lesion compared to those made in the present study. On the other hand, TEMDP lesions produced here were similar to those found in a recent study [11].

Lastly, a Buffer lesion was produced following previous studies [4, 14, 22–24]. This protocol seemed to be well reproducible compared to the results of Magalhães et al. [4] and Queiroz et al. [24]. Furthermore, Buffer solution is one of the most useful protocol among the researchers to induce artificial caries-like lesion [4, 14, 22–25], which does not mean that this protocol reproduce better the oral environment compared to the others.

TMR is considered the gold standard technique for measuring differences in mineral content of subsurface dental lesions [26]; however, differences between the TMR systems might be responsible for the disparities in the reproducibility among the demineralising protocols worldwide [17]. On the other hand, cross-sectional hardness is an alternative method to measure demineralisation with some limitations, as discussed elsewhere [4].

TMR and cross-sectional hardness data presented a strong correlation and a linear relationship between them for both demineralised and de-remineralised enamel. Therefore, the null hypotheses number 3 and 4 were rejected. Although the methods presented a good correlation, they have distinct sensitivity to detect the remineralising response of different lesions to the tested treatments.

Considering the hardness data (Table 2), the Buffer and PA lesions showed the highest remineralisation while TEMDP showed the worst remineralising response. Furthermore, no dose–response was found for TEMDP (SH and 10 μm CSH) and MC gel (10 μm CSH). Oppositely, despite no difference was found among the lesions for TMR (Table 4), Buffer lesion presented a negligible remineralisation as shown in Fig. 2. This result is quite interesting, as no previous study has applied both methods to compare the response of four artificial caries lesions to three remineralising treatments. From these results, hardness seems to be a sensitive method to analyse remineralisation of shallow and less demineralised lesions, while TMR is more indicated for deep and highly demineralised lesions.

Generally, studies have shown that highly demineralised and deep lesions remineralise more than shallow lesions using TMR system [2, 7, 9, 27, 28]. This tendency was seen for TMR analysis, only in the absence of fluoride (Table 4). This result might be explained by the low mineral content in the lesion body, allowing high mineral uptake from saliva. As fluoride acts more on the lesion surface, this tendency was not seen under the fluoride regimens. Considering hardness (Table 2) and TMR (only for the control phase, Table 4), we rejected the null hypothesis number 1.

Other important factor is the presence of a pseudo-intact surface layer [4]. It has been shown that lesions with a less mineralised surface layer react better to fluoride at the surface [18], in agreement with our results. In our study, PA gel and Buffer showed some superficial remineralisation (Table 2) by the use of fluoride, probably due to the presence a porous surface. Accordingly, differences among the remineralising treatments were found only for the analyses of the surface hardness. Considering previous *in vitro* models, the protocol that has shown the best dose–response to the remineralising effect of fluoride is the Buffer lesion [25, 29, 30], by using hardness analysis.

Since we found differences between both fluoride treatments and control (for hardness analysis), the hypothesis number 2 can be rejected. However, our study did not show any further effect of the combination of professional plus home-care fluoride application compared to home-care fluoride treatment only. Our finding is in agreement with Lagerweij & ten Cate [31]. They found a reduction in enamel lesion size of 54 % in the 1450 ppm F toothpaste + 12500 ppm F gel group (home-care plus professional treatment) and of 44 % in the 1450 ppm F toothpaste-only group (home-care treatment), with no difference between both, after 4 weeks of daily treatment. According to the authors, the enhancement of remineralisation is positively but not linearly related to the concentration of fluoride, especially when high fluoride concentrations are used.

One hypothesis to explain the lack of difference might be the absence of cariogenic challenges *in situ*. In the absence of dental biofilm and sucrose exposure, which are able to reduce the pH surround the tooth, the F^−^ enamel reservoir (like-CaF_2_) produced by the varnish could not interact with the deeper part of the lesions, not allowing a better remineralising effect of the combined treatments compared to the home-care fluoride treatment only.

In a previous study, a modest remineralisation by the use of fluoride dentifrice was seen in high R lesions (highly demineralised lesions), even after 3–4 weeks of *in situ* model. On the other hand, low R lesions presented a slight demineralisation after 3 weeks. Both lesions were exposed to the oral cavity in the presence of biofilm, but without cariogenic challenges, *in situ* [5]. A similar modest remineralising effect was shown in the *in situ* study of Comar et al. [11]*,* where TEMDP lesions were treated with fluoride varnish [11]. Therefore, we speculate that rather than remineralising time (>3 days) or/and the presence of biofilm, there is a need of including cariogenic challenges to allow detection of a dose–response effect of fluoride, especially when high fluoride concentrations are tested.

Some insight into the remineralisation process was previously discussed. Tschoppe and Meyer-Lueckel [9] only found a dose–response relationship in the remineralising effect of fluoride dentifrices when demineralised enamel was exposed to saliva substitutes that induced demineralisation rather than remineralisation. Therefore, it may be necessary to intercalate demineralisation between remineralisation for better detection of the lesion’s response to different fluoride sources, especially for high concentrated fluoride products that are usually applied only at once (E.g. varnish).

We suggest that further similar studies shall be done, with biofilm accumulation and different cariogenic challenges (varying the frequency of sucrose exposure), to mimic patients with different caries activities, in order to confirm this hypothesis for the combination of fluoride varnish and toothpaste. Another important aspect for further consideration is whether a single (or more) application(s) of fluoride varnish is (are) needed to improve enamel remineralisation compared to the daily use of fluoride dentifrice using long-term in situ models.

## Conclusion

Based on the present data and considering the limitations of the study, the most responsive lesions to fluoride were Buffer and PA gel. For these less demineralised lesions, hardness should be the choice as response variable. On the other hand, MC gel and TEMDP lesions might better simulate the lesions for those the clinicians would indicate professional fluoride treatment. For these highly demineralised lesions, fluoride was ineffective to improve remineralisation under this *in situ* protocol. Further *in situ* studies must be done, including biofilm and cariogenic challenges, in order to check the ability of those lesions to respond to different fluoride regimens.

The findings highlight the importance of carefully inferring data from *in vitro* studies, in which only a type of lesion is applied to test a new remineralising product. Therefore, this study suggests that more than one type of lesion (slightly and highly demineralised ones) should be included in further studies to better understand the remineralising effect of a new product, considering the presence of more than one response variable.

## Abbreviations

MC: methylcellulose
PA: polyacrylic acid
TEMDP: tetraethyl methylene diphosphanate
C: Control
F^−^: Fluoride
FD: Fluoride dentifrice
FVD: fluoride varnish and dentifrice
TMR: transverse microradiography
SH: surface Hardness
SHC: surface hardness change
SHR: surface hardness recovery
CSH: cross-sectional hardness
R: average mineral loss
LD: lesion depth
ΔZ: integrated mineral loss
SL: surface layer
S: Surface
ΔZR: integrated mineral loss recovery
%CSHR: percentage of cross-sectional hardness recovery

## References

[1] Arends J, Dijkman T, Christoffersen J (1987). Average mineral loss in dental enamel during demineralization. Caries Res.

[2] Lynch RJ, Ten Cate JM (2006). The effect of lesion characteristics at baseline on subsequent de- and remineralisation behaviour. Caries Res.

[3] Yamazaki H, Margolis HC (2008). Enhanced enamel remineralization under acidic conditions in vitro. J Dent Res.

[4] Magalhães AC, Moron BM, Comar LP, Wiegand A, Buchalla W, Buzalaf MAR (2009). Comparison of Cross-Sectional Hardness and Transverse Microradiography of Artificial Carious Enamel Lesions Induced by Different Demineralising Solutions and Gels. Caries Research.

[5] Lippert F, Lynch RJ, Eckert GJ, Kelly SA, Hara AT, Zero DT (2011). In situ fluoride response of caries lesions with different mineral distributions at baseline. Caries Res.

[6] Lippert F (2012). The effects of lesion baseline characteristics and different Sr:Ca ratios in plaque fluid-like solutions on caries lesion de- and remineralization. Arch Oral Biol.

[7] Lynch RJ, Mony U, ten Cate JM (2007). Effect of lesion characteristics and mineralizing solution type on enamel remineralization in vitro. Caries Res.

[8] González-Cabezas C, Jiang H, Fontana M, Eckert G (2012). Effect of low pH on surface rehardening efficacy of high concentration fluoride treatments on non-cavitated lesions. J Dent.

[9] Tschoppe P, Meyer-Lueckel H (2012). Effects of regular and highly fluoridated toothpastes in combination with saliva substitutes on artificial enamel caries lesions differing in mineral content. Arch Oral Biol.

[10] Buchalla W, Imfeld T, Attin T, Swain MV, Schmidlin PR (2008). Relationship between nanohardness and mineral content of artificial carious enamel lesions. Caries Res.

[11] Comar LP, Wiegand A, Moron BM, Rios D, Buzalaf MA, Magalhães AC (2012). In situ effect of sodium fluoride or titanium tetrafluoride varnish and solution on carious demineralization of enamel. Eur J Oral Sci..

[12] Delbem AC, Danelon M, Sassaki KT, Vieira AE, Takeshita EM, Brighenti FL (2010). Effect of rinsing with water immediately after neutral gel and foam fluoride topical application on enamel remineralization: An in situ study. Arch Oral Biol.

[13] Klimek J, Hellwig E, Ahrens G (1982). Fluoride taken up by plaque, by the underlying enamel and by clean enamel from three fluoride compounds in vitro. Caries Res.

[14] Magalhães AC, Comar LP, Rios D, Delbem AC, Buzalaf MA (2008). Effect of a 4 % titanium tetrafluoride (TiF_4_) varnish on demineralisation and remineralisation of bovine enamel in vitro. J Dent..

[15] Angmar B, Carlstrom D, Glas JE (1963). Studies on the ultrastructure of dental enamel. IV. The mineralization of normal human enamel. J Ultrastruct Res.

[16] ten Cate JM (1994). In situ models, physico-chemical aspects. Adv Dent Res.

[17] ten Cate JM, Dundon KA, Vernon PG, Damato FA, Huntington E, Exterkate RA (1996). Preparation and measurement of artificial enamel lesions, a four-laboratory ring test. Caries Res.

[18] Lippert F, Butler A, Lynch RJ, Hara AT (2012). Effect of fluoride, lesion baseline severity and mineral distribution on lesion progression. Caries Res.

[19] White DJ (1987). Use of synthetic polymer gels for artificial carious lesion preparation. Caries Res.

[20] Iijima Y, Cai F, Shen P, Walker G, Reynolds C, Reynolds EC (2004). Acid resistance of enamel subsurface lesions remineralized by a sugar-free chewing gum containing casein phosphopeptide-amorphous calcium phosphate. Caries Res.

[21] Buskes JA, Christoffersen J, Arends J (1985). Lesion formation and lesion remineralization in enamel under constant composition conditions. A new technique with applications. Caries Res.

[22] ten Cate JM, Duijsters PP (1983). Influence of fluoride in solution on tooth demineralization. I. Chemical data. Caries Res..

[23] ten Cate JM, Duijsters PP (1983). Influence of fluoride in solution on tooth demineralization. II. Microradiographic data. Caries Res..

[24] Queiroz CS, Hara AT, Paes Leme AF, Cury JA (2008). pH-cycling models to evaluate the effect of low fluoride dentifrice on enamel de- and remineralization. Braz Dent J.

[25] Gomez J, Pretty IA, Santarpia RP, Cantore B, Rege A, Petrou I (2014). Quantitative Light-Induced Fluorescence to Measure Enamel Remineralization in vitro. Caries Res.

[26] Clasen AB, Ogaard B (1999). Experimental intra-oral caries models in fluoride research. Acta Odontol Scand.

[27] Strang R, Damato FA, Creanor SL, Stephen KW (1987). The effect of baseline lesion mineral loss on in situ remineralization. J Dent Res.

[28] Iijima Y, Takagi O, Ruben J, Arends J (1999). In vitro remineralization of in vivo and in vitro formed enamel lesions. Caries Res.

[29] Lippert F, Newby EE, Lynch RJ, Chauhan VK, Schemehorn BR (2009). Laboratory assessment of the anticaries potential of a new dentifrice. J Clin Dent.

[30] Hirata E, Danelon M, Freire IR, Delbem AC (2013). In vitro enamel remineralization by low-fluoride toothpaste with calcium citrate and sodium trimetaphosphate. Braz Dent J.

[31] Lagerweij MD, ten Cate JM (2002). Remineralisation of enamel lesions with daily applications of a high-concentration fluoride gel and a fluoridated toothpaste: an in situ study. Caries Res.

